# Multilingualism and international mental health research–The barriers for non-native speakers of English

**DOI:** 10.1371/journal.pmen.0000033

**Published:** 2024-06-04

**Authors:** Giovanni de Girolamo, Stefano Calza, Massimo Clerici, Armando D’Agostino, Diana De Ronchi, Andrea Fagiolini, Giuseppe Nicolò, Maria Nobile, Andrea Raballo, Paola Rucci, Alessandro Serretti, Fabrizio Starace, Pietro Tiraboschi, Paolo Brambilla

**Affiliations:** 1 IRCCS Istituto Centro San Giovanni di Dio Fatebenefratelli, Unit of Epidemiological Psychiatry, Brescia, Italy; 2 Department of Molecular and Translational Medicine, Unit of Biostatistics and Bioinformatics, University of Brescia, Brescia, Italy; 3 Department of Mental Health and Addiction, Section of Psychiatry, School of Medicine and Surgery, University of Milan Bicocca, Milan, Italy; 4 Fondazione IRCCS San Gerardo dei Tintori, Monza, Italy; 5 Department of Health Sciences, Section of Psychiatry, University of Milan, Milan, Italy; 6 Department of Biomedical and Neuromotor Sciences, Section of Psychiatry, University of Bologna, Bologna, Italy; 7 Department of Molecular and Developmental Medicine, Section of Psychiatry, University of Siena, Siena, Italy; 8 Department of Mental Health, Rome, Italy; 9 Child Psychopathology Unit, IRCCS Eugenio Medea, Bosisio Parini, Italy; 10 Faculty of Biomedical Sciences, Department of Health, and Social Care, Chair of Psychiatry, Università della Svizzera Italiana, Lugano, Switzerland; 11 Public Health Division, Cantonal Socio-Psychiatric Organization, Repubblica e Cantone Ticino, Mendrisio, Switzerland; 12 Department of Biomedical and Neuromotor Sciences, Unit of Public Health, Biostatistics and Health Services Research, University of Bologna, Bologna, Italy; 13 Department of Medicine and Surgery, Kore University of Enna, Enna, Italy; 14 Department of Mental Health and Pathological Addiction, AUSL di Modena, Modena, Italy; 15 Neurology V and Neuropathology Unit, Fondazione IRCCS Istituto Neurologico Carlo Besta, Milan, Italy; 16 Department of Pathophysiology and Transplantation, Section of Psychiatry, University of Milan, Milan, Italy; PLOS: Public Library of Science, UNITED KINGDOM

In 2023, an international mental health congress was held in Italy. Although this meeting was attended by 1,389 participants from all over the world, there was only an estimated 54 (3.8%) Italian participants. This could be considered to be very low attendance of Italian scientists and clinicians for an event of this size taking place in their country of residence.

We believe that the language barrier is a principal reason for such a low attendance. According to the *English Proficiency Index* developed by Education First [1], Italy ranks 35th out of 112 countries surveyed in terms of English proficiency: other European countries rank similarly low in this list (Spain is 35^th^, France is 43^rd^); whilst other large countries outside of Europe also place low in this ranking (Brazil 70^th^, Indonesia 79^th^, China 82^nd^, Japan 87^th^,) as do most low-income countries.

Limitations in English fluency are also present among Italian medical professionals, which could strongly inhibit their capacity to actively participate in meetings held entirely in English: in Italy the first university medical course taught entirely in English was held in Pavia in 2009, and since then other courses have been started, but they involve a minority of all medical students enrolled every year: for instance, in 2023 on a total of 18,249 first-year medical students, those enrolled in Englih-speaking courses were only 1,779 (9.6%) [2].

There are over 7,000 living languages in the world today [3], and English is only one of them. At the time of writing, there are 373 million native speakers (who speak English as their first language), whilst 1,080 million speak English as their second language [3]. With a current world population estimated by the United Nations at 7,954 billion [4], this means that native English speakers make up approximately 4.7% of the world’s population: meaning that an overwhelming majority speaks a different native language. Gordin [5], Professor of modern and contemporary history at Princeton University, has commented that “*There’s nothing about English that makes it intrinsically better for science than any other language*. *Science could have gone just as far in Chinese or Swahili*. *But many economic and geopolitical forces made English the dominant language of research*, *for better or worse*”. Godin’s statement underscores a crucial aspect of the contemporary landscape of scientific and mental health communication and research dissemination: the dominance of English in the scientific and mental health community is not a consequence of its intrinsic qualities as a language, but rather the result of historical, economic, and geopolitical developments. This dominance has far-reaching implications for the global scientific and mental health community, particularly for non-native English speakers. It highlights a systemic barrier that can hinder access to scientific knowledge, including advancements in mental health research, contribute to inequalities in academic publishing, and limit the participation of researchers from diverse linguistic backgrounds in the global discourse. The acknowledgment of English’s dominance due to external forces rather than inherent superiority calls for a critical examination of current practices and encourages efforts to make scientific and mental health communication more inclusive. This inclusivity could involve supporting multilingual publications, providing translation services, and fostering a scientific and mental health culture that values and accommodates linguistic diversity.

Native speakers who grew up in an English-speaking country typically have a vocabulary size of 15,000-to-20,000-word families—or lemmas [6]. In contrast, non-native speakers typically have a vocabulary of only 2,000 to 3,000 words. However, while reading and writing in a non-native language can allow opportunities to translate and revise what is read or what is written, this is more difficult for spoken language. This constitutes the main barrier for communication at meetings–attending oral presentations in someone else’s native language poses a massive disadvantage for people who must make a considerable additional effort to internally translate that language. Moreover, while recent systems of AI can greatly help in written English [7], this option does not apply as widely to spoken English. For all these reasons, the language barrier represents a primary source of exclusion—in this case from active participation in a mental health research community setting. However, as noted by Amano et al. (2021) [8], “*Less fluent language skills*, *however*, *do not equate to poorer quality of science*”. We have commonly observed that many English-native scientists and clinicians speak very fast, use local jargon or dialects, or choose complex syntactic sentences when delivering talks or when engaging in personal conversation with non-native colleagues. In summary, many seem to be unaware of the cognitive difficulties faced by colleagues who are not native speakers of English. Understanding and acknowledging the challenges that individuals face when communicating in a language different from their mother tongue is crucial, especially for mental health professionals. Communication is a fundamental aspect of human connection, and for mental health practitioners, the ability to comprehend and navigate the intricacies of communication is central to their role and understanding their patients and each other. The paradox lies in the fact that while these professionals are dedicated to understanding and addressing the personal and often highly complex and multifactorial difficulties of their clients, language barriers can inadvertently hinder effective communication. When individuals express themselves in a language that is not their primary one, it introduces a layer of complexity that can obscure the true nature of their thoughts, emotions, and experiences. Miscommunications may arise, leading to potential misunderstandings or incomplete assessments of a person’s mental well-being.

It is important at this point to acknowledge that language barriers present a significant global challenge that affects scientists and clinicians from every continent and country, not just those in Europe. This issue spans across diverse linguistic backgrounds, creating obstacles for non-native English speakers worldwide in accessing, contributing to, and engaging with international research. These barriers not only limit the dissemination of scientific knowledge, including advancements in mental health research, but also restrict the exchange of ideas and innovations.

Given this situation and acknowledging the role of English as the official language of the international scientific and mental health community, we wish to make a few suggestions which can be easily used by our native English-speaking colleagues attending international meetings; we also encourage the use of English in medical schools, which will contribute to the professional development of health workers in a globalized world. Several general tips have been proposed in the past for overcoming language barriers (oral or written) in all science [8]. We feel that four simple rules do not imply any special effort by English-native colleagues, but they can greatly facilitate spoken communication with non-native speakers of English and foster fuller participation of all colleagues in international mental health meetings and congresses:

1. *Speak slowly*, avoiding excessive speed which may make the talk very difficult to understand for colleagues with reduced English fluency.
2. *Avoid the use of local dialects*, *slang or informal vocabulary* which can considerably increase difficulties in understanding the content of a talk.
3. *Simplify the syntactic structure of sentences*: even the most complex topics can be expressed in simple ways, and this can certainly facilitate understanding for all.
4. *Make an effort* to produce slides which facilitate understanding by non-native people, with clear and brief sentences, avoiding uncommon acronyms, etc.

Compliance with these simple rules will not hamper the quality and the richness of scientific content, including advancements in mental health reserch, that can be disseminated, but it will reduce a major source of disparity, increase accessibility and facilitate the sharing of knowledge with colleagues who were simply born in a country where one of the other 7,167 other languages in the world is spoken.
